# Supplementary material for: Isolation and Functional Analysis of MbCBF2, a Malus baccata (L.) Borkh CBF Transcription Factor Gene, with Functions in Tolerance to Cold and Salt Stress in Transgenic Arabidopsis thaliana
Source: Int J Mol Sci. 2022 Aug 29;23(17):9827. doi: 10.3390/ijms23179827 (PMC9456559; doi:10.3390/ijms23179827)
Supplement: Supplementary file 1 [file ijms-23-09827-s001.zip › ijms-1878088-supplementary.pdf]

Table S1. Primers used in this study

Primer Name	Primer sequence (5'→3')	Function
MbCBF2-F	ATGGATGCTTTCTCTCATTA	Clone full length of *MbCBF2*
MbCBF2-R	CATTATGGAGTTTTTCCATTTGA	Clone full length of *MbCBF2*
q-PCR-F	CTCGCCCCATGTTCATGAACTTGT	q-PCR for *MbCBF2*
q-PCR-R	GCGGCCATTTCTGCCGTGGGAAAA	q-PCR for *MbCBF2*
Actin-F	ACACGGGGAGGTAGTGACAA	q-PCR for Actin
Actin-R	CCTCCAATGGATCCTCGTTA	q-PCR for Actin
AtCOR15a-F	CAACAGAGGAATCACCAGCGA	Clone full length of *AtCOR15a*
AtCOR15a-R	CTCTGCTGTCTTGTCGTGGTGT	Clone full length of *AtCOR15a*
AtRD29a-F	CAACGAGGGGAAGATAAAAGTGT	Clone full length of *AtRD29a*
AtRD29a-R	AGCCAGATGATTTTGGAGCCT	Clone full length of *AtRD29a*
AtCOR6.6-F	ACAGGCGGGAAAGAGTAT	Clone full length of *AtCOR6.6*
AtCOR6.6-R	TGGAAGGCATTCTTGTTG	Clone full length of *AtCOR6.6*
AtERD10-F	GCAGCAGGAGGAGAAGGG	Clone full length of *AtERD10*
AtERD10-R	CACCAGGAAGAAGCCCATC	Clone full length of *AtERD10*
AtCOR47-F	TGGTTGTAACGGAGCATC	Clone full length of *AtCOR47*
AtCOR47-R	CCCCAAGAAATCAAACAA	Clone full length of *AtCOR47*
AtRD29b-F	CAACGAGGGGAAGATAAAAGTGT	Clone full length of *AtRD29b*
AtRD29b-R	AGCCAGATGATTTTGGAGCCT	Clone full length of *AtRD29b*
AtNCED3-F	ATGGCTTCTTCACGGCACGG	Clone full length of *AtNCED3*
AtNCED3-R	TTCCTTTGCCCTCGGACG	Clone full length of *AtNCED3*
AtCAT1-F	CGCCATGCCGAAAAATACCC	Clone full length of *AtCAT1*
AtCAT1-R	CTTGCCTGTCTGAATCCCAGGAC	Clone full length of *AtCAT1*
AtP5CS-F	GATACGGATATGGCAAAGCG	Clone full length of *AtP5CS*

AtP5CS-R	CCAAGTCCAAATCGGAAACC	Clone full length of *AtP5CS*
AtPIF1-F	ATTTCCGAGCACAGAGTAGA	Clone full length of *AtPIF1*
AtPIF1-R	TCTACTCTGTGCTCGGAAAT	Clone full length of *AtPIF1*
AtPIF4-F	GCATCACAACCGACCGTAAG	Clone full length of *AtPIF4*
AtPIF4-R	ATTCCACTCCCCATCCACAT	Clone full length of *AtPIF4*
AtSnRK2.4-F	GAGGAAATGGGGATGCAGAT	Clone full length of *AtSnRK2.4*
AtSnRK2.4-R	TTCTCACTTCTCCACTTGCG	Clone full length of *AtSnRK2.4*

ATGGATGCTTTCTCTCATTACTACGTGGACCCGCAGCCGGAATCATCGTCCGCTTCTGACGCCAACAGTTCTCGCCCCATGTTCATGAACTTGTCG
M D A F S H Y Y V D P Q P E S S S A S D A N S S R P M F M N L S
GACGAGGAGGTCCTGCTGGCGTCCACTTACCCGAAGAAGCAGGCTGGACGGAAGAAGTTTAAAGAGACAAGGCACCCTGTGTACCGCGGTGTCCGG
D E E V L L A S T Y P K K Q A **G R K K F K E T R H P V Y** R G V R
AGGAGGAACTCCGGCAAGTGGGTGTCCGAGGTCCGGGAGCCCAACAAAAAGACAAGGATTTGGCTGGGAACATTTCCCACAGCCGAAATGGCCGCG
R R N S G K W V S E V R E P N K K T R I W L G T F P T A E M A A
CGCGCTCATGATGTGGCGGCCATTGCGCTGAGGGGGAGGTCGGCTTGCCTCAACTTTGCCGACTCTGCGTGGCGGCTACCAGTCCCTGCCTCCGCC
R A H D V A A I A L R G R S A C L N F A D S A W R L P V P A S A
AACGCGAAGGATATTCAAACTGCCGCGGCGGAAGCAGCAGAACAGTTTCGGCCGGTGGAAGAGTCGGATGAGGCTTGTGGTGCTTCTGCGGTGGCT
N A K D I Q T A A A E A A E Q F R P V E E S D E A C G A S A V A
CCCTCGGAGTGCACGTCAGCGTCGGAGCAACAAGTGCATTTTATGGATGAGGAAGCGGTTTTTGGGATGCCGGGACTGCTGGCAAATATGGCTGAA
P S E C T S A S E Q Q V H F M D E E A V F G M P G L L A N M A E
GGAATGCTACTGCCTCCACCTCATTACAGTGACGATGATGACGTGTATGTTTATGCTGACGTGCCATTATGGAGTTTTTCCATTTGA
G M L L P P P H Y S D D D D V Y V Y A D V P L W S F S I *

Figure S1. The predicted amino acid sequence and nucleotide of *MbCBF2* gene.
